# ACD/Structure Elucidator: 20 Years in the History of Development

**DOI:** 10.3390/molecules26216623

**Published:** 2021-11-01

**Authors:** Mikhail Elyashberg, Antony Williams

**Affiliations:** 1 Advanced Chemistry Development, Moscow Department, 6 Akademik Bakulev Street, 117513 Moscow, Russia; 2 ChemConnector, 513 Chestnut Grove Court, Wake Forest, NC 27587, USA

**Keywords:** Nuclear Magnetic Resonance (NMR), computer-assisted structure elucidation (CASE), structure elucidation, NMR prediction

## Abstract

The first methods associated with the Computer-Assisted Structure Elucidation (CASE) of small molecules were published over fifty years ago when spectroscopy and computer science were both in their infancy. The incredible leaps in both areas of technology could not have been envisaged at that time, but both have enabled CASE expert systems to achieve performance levels that in their present state can outperform many scientists in terms of speed to solution. The computer-assisted analysis of enormous matrices of data exemplified 1D and 2D high-resolution NMR spectroscopy datasets can easily solve what just a few years ago would have been deemed to be complex structures. While *not* a panacea, the application of such tools can provide support to even the most skilled spectroscopist. By this point the structures of a great number of molecular skeletons, including hundreds of complex natural products, have been elucidated using such programs. At this juncture, the expert system ACD/Structure Elucidator is likely the most advanced CASE system available and, being a commercial software product, is installed and used in many organizations. This article will provide an overview of the research and development required to pursue the lofty goals set almost two decades ago to facilitate highly automated approaches to solving complex structures from analytical spectroscopy data, using NMR as the primary data-type.

## 1. Introduction

The first publications devoted to the development of CASE systems (Computer-Assisted Structure Elucidation) appeared more than 50 years ago [1,2,3,4], and it is now a well-known approach in the chemical community. Currently, there are several expert systems (ES) [5,6,7,8,9,10,11] available for the computerized elucidation of small molecule structures from solution-state NMR data. Among these systems, ACD/Structure Elucidator (ACD/SE) is, probably, the most advanced and certainly the most well documented in terms of scientific publications and validation studies [12,13,14,15]. The numerous statistics representing improved performance over the many years of development, and the myriad examples demonstrating the gradual increase in the complexity of structures that could be solved using the system, are reported elsewhere [16]. The authors of this article were both early members of the team that initiated the work and were key in the development of the ACD/SE system from its inception. One of us (ME) remains an active member of the team to date while the other (AJW) pursued the application of cheminformatics approaches to deliver data to the community via open websites yet, as an NMR spectroscopist, still celebrates the decade of work on the system as one of the highlights of his career. Here we collectively bring our knowledge of the system to bear on defining the research path that directed the development of this system and, even today, continues to progress in capability. 

## 2. ACD/SE First Version: An Expert System Based on 1D NMR Spectra

**Figure 1:**
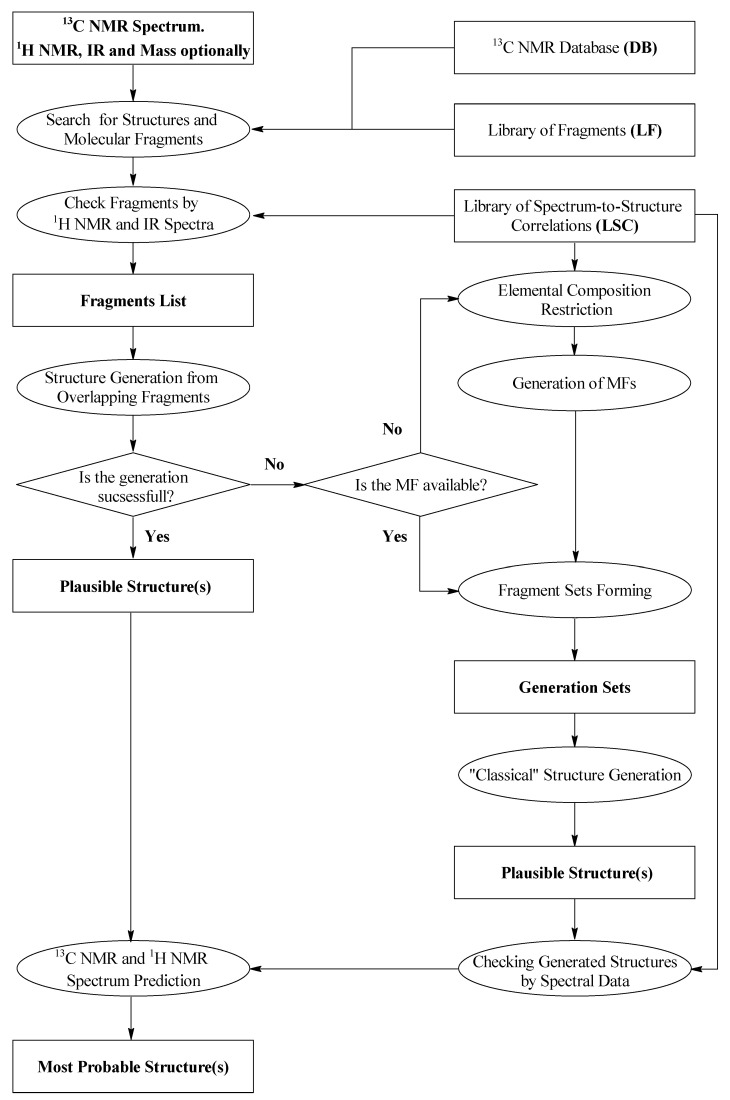
Flow diagram of ACD/SE_v1 based on 1D ^13^C NMR spectra. During the first stage, the system tried to automatically determine the structure of the unknown compound using fragments found as a result of the Library of Fragments search based on the ^13^C NMR spectrum. The resultant structure set was then generated by overlapping and joining fragments that had *common atoms*. Knowledge regarding the molecular mass and the molecular formula were not needed for this stage.

An initial exploration of expert systems based only on fragment libraries containing small substructures and their associated characteristic features in ^13^C, ^1^H, and IR spectra [17,18] showed that only relatively simple structures of a modest size (10–20 skeletal atoms) could be elucidated using this approach. An attempt to create libraries containing fragments of larger size characteristic of specific chemical classes turned out to not be practical because an ES loses its general universality in such a case. Will et al. [19] suggested that a database of ca. 500,000 fragments associated with their ^13^C NMR sub-spectra would be required to deal with chemicals of even medium complexity. Such a database was created from a seed set containing over 200,000 organic molecules and the authors [19] reported that the structure of ~80% of all pure organic compounds with a molecular weight ≤ 1000 measured routinely in main BASF laboratory could be resolved automatically with this software. Using this work as the basis of our first development, we developed a new hybrid expert system, ACD/SE_v1 supported by *two* structure generators [20]. The knowledgebase underpinning this first version of ACD/SE was generated from a database containing ~135,000 structures and associated ^13^C NMR spectra to produce a resulting library of molecular fragments and more than 400,000 ^13^C NMR sub-spectra and a library of spectrum-to-structure correlations for both NMR and IR spectra totaling ca. 450 substructures. A flow diagram describing ACD/SE_v1 is shown in Figure 1.

If this approach failed, then the program automatically determined the molecular formula from the molecular mass and then formed sets of fragments. Note, however, that different formulae can have the same mass depending on the accuracy of determination of the mass. Structures were generated with the help of a “classical” generator [21] designed to assemble structures from *non-overlapping* fragments. The libraries of spectrum-to-structure correlations (LSC) were used to determine the molecular formula as well as for spectral filtration of the fragments and structures. The most preferable structure was selected by predicting both the ^13^C and ^1^H NMR spectra using ACD/NMR predictors which had been developed previously and were integrated into ACD/SE. At that time the program ran on an IBM PC computer (133 MHz) operating under Windows 3.x or Windows 95/98. 

The performance of the system was initially tested on a set of 220 problems obtained from student textbooks and spectrum collections. The validation test showed that in 90% of cases the correct solution was found, and the resultant structures were molecules of small to medium sizes (up to 20–25 heavy atoms). The system, however, demonstrated failure when no fragments corresponding to a relatively large fragment of the molecule were found. 

## 3. ACD/SE Second Version: An Expert System Based on 1D and 2D NMR Data 

**Figure 2:**
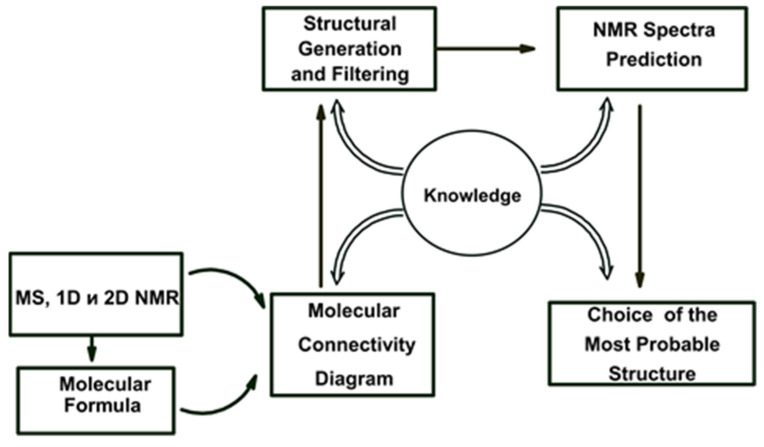
A simplified flow diagram for ACD/SE_v2 based on 1D and 2D NMR spectra.

The chemistry of natural products generally deals with large molecules frequently containing 25–100 skeletal atoms, and ACD/SE_v1 was generally destined to fail for the identification of chemicals of this complexity. Based on our previous experiences, and those reported in the literature [22,23,24,25], we extended the system to support multiple types of 2D NMR spectra, specifically ^1^H-^1^H, ^1^H -^13^C and ^1^H-^15^N NMR experiments in combination with a knowledgebase supporting the structure elucidation of larger molecules represented by natural products [26]. Many new features were incorporated into this system to facilitate ACD/SE supporting the structural interpretation of 2D NMR spectra. A simplified flow diagram of ACD/SE_v2 is shown in Figure 2.

Possible data that could be utilized in v2 were a molecular formula, ^1^H & ^13^C 1D NMR, and ^1^H-^1^H COSY, ^1^H-^13^C HSQC (or HMQC), ^1^H-^13^C HMBC and ^1^H-^15^N HMBC 2D NMR (if available). Processing of the spectral data was carried out using the integrated ACD/SpecManager module, which imported the spectral data and assembled the data into tables of chemical shifts, multiplicities (if determined) and intensities of signals for 1D NMR spectra. The chemical shifts of coupled nuclei and peak intensities were extracted from the relevant 2D data into a set of resulting tables including, when data were available, the C-C and C-N connectivities with the following bond distances between coupling nuclei in the 2D NMR spectra set as *default*: ^1^H-^1^H COSY (2–3 bonds for strong peaks and 3–4 bonds for weak ones) and HMBC (2–3 bonds). The connectivity tables could be refined, if necessary, using user interpretation of the 2D peak intensities.

**Figure 3:**
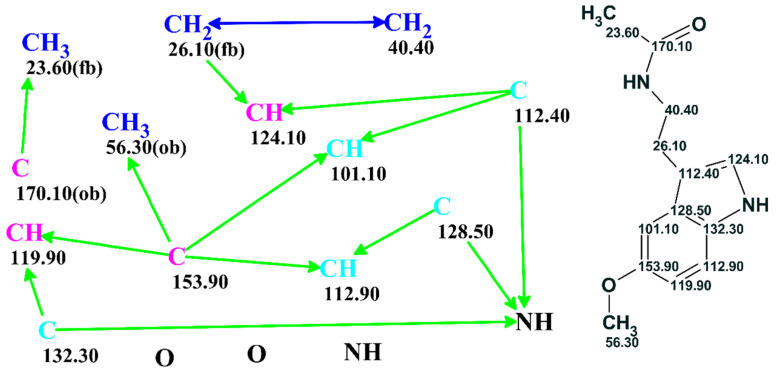
MCD created for the structure shown on the right using data obtained from both HMBC and COSY spectra. Carbon atom hybridization is marked in color as: *sp*^3^—blue, *sp*^2^—violet, *not sp* (*sp*^3^ or *sp*^2^)—light blue. HMBC connectivities are indicated by green arrows and COSY connectivities by blue arrows. The label “*fb*” near a carbon atom means that bonding with a heteroatom is forbidden, while the label “*ob*” means that bonding of that given atom to at least one heteroatom is obligatory. The MCD was first introduced ACD/SE_v2 and remains important to present day as a visual cue of NMR correlations.

The problem could be further processed either automatically or under user supervision. A full picture of the skeletal atom properties and connectivities could be visualized as a “Molecular Connectivity Diagram” (MCD) as exemplified in Figure 3. The program lays out the quaternary C, CH, CH2 and CH3 groups as well as free hydrogen atoms, if they exist, and all available heteroatoms provided via the molecular formula in the display window. Connectivities existing between atoms are visualized and colored in different colors for COSY (blue) and HMBC (green) correlations. In addition, the chemical shifts of the individual C, N and H atoms were displayed. At this stage, the connectivity distances could be edited, and chemical bonds directly input by the user based on prior information if available. 

The structure generation process [27,28] speeds up dramatically, and the number of possible structures is reduced, if the atom hybridization states and the possibility of having neighboring heteroatoms are considered. With this in mind, we automatically formed a ^13^C NMR correlation table from the system knowledgebase (~135,000 structures). The table contains carbon atom-centered fragments (ACFs) with the corresponding intervals of chemical shift variation for the central carbon atom. The program uses this ACF table for the automatic assignment of the hybridization (*sp*^3^*, sp*^2^*, sp, not sp, not defined*) to all carbons and for assessing the possibility of their neighboring heteroatoms (forbidden—*fb*, obligatory *ob*, not defined). The correlation table is referred as the Atom Property Correlation Table (APCT). The properties assigned and the associated connectivities could be edited by the user.

If the 2D NMR data are consistent (all HMBC and COSY correlations are as default, *^n^J*_CH,HH_, *n* = 2–3), then it was expected that the solution would be *valid* (that is, the output file would contain the correct structure). The initial data became *contradictory* if those correlations whose length *n* > 3 (referred to as *non-standard correlations*, NSCs) occurred in the connectivity tables. For example, if the distance between two coupled nuclei is equal to 4 bonds but is specified as 2–3 bonds in the table (and in the MCD), then the correct structure will be absent in the results file. Therefore, prior to starting structure generation the program carried out an automatic check of the connectivity tables and associated MCD for the presence of contradictions. The check was based on logical analysis of the cumulative 2D NMR data. If contradictions were detected, then the program displayed a message that enumerated the contradictions detected and explained their causes. The carbon atoms which were assigned with potential connectivities classed as *^n^J_HH_* or *^n^J_CH_* (where *n* = 4) were detected, then *all* connectivities associated from these atoms were elongated by one bond during structure generation (that is *n* = 2–4 instead of *n* = 2–3). With this approach, if the real length *n* of a given connectivity was *n >* 4 then the described approach would not help and the solution to the problem would be *invalid*. Nevertheless, this method allowed for the detection of the presence of NSCs in 2D NMR data and should be considered as a very important result.

To select the most probable structure as the result, the ACD\CNMR Predictor software program was used. The ^13^C NMR chemical shifts for all structures were calculated using an *incremental* approach [13], and the structures were rated by the average deviation (*d_F_*) of the calculated spectrum from the experimental one. The assignment of estimated shifts to the observed ones was performed automatically and the structures were ranked in order of increasing average deviations, with the top ranked structure considered as the most probable match.

**Figure 4:**
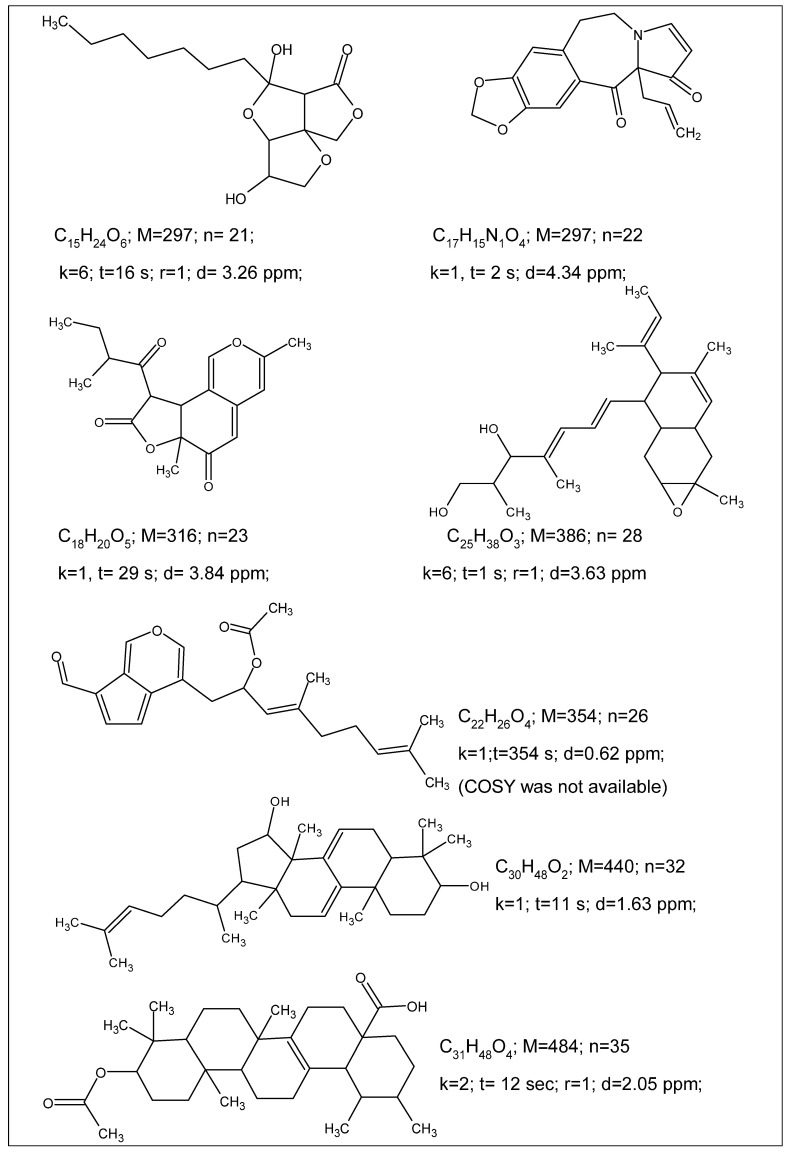
Examples of structures elucidated during multiple test runs of ACD/SE_v2. In the reported parameters, *n* is the number of heavy atoms in a molecule, *k* is the number of found candidate structures, *t* is the generation time in seconds on a 500 MHz PC Pentium, *r* is the position of the real structure in the ranked structural file and *d* is the average deviation of the ^13^C experimental spectrum relative to the predicted spectrum.

The ACD/SE_v2 program was tested multiple times using 2D NMR data published in multiple articles devoted to natural product structure elucidation. In so doing, we manually introduced the connectivities presented in the tables which were reported by the authors of the corresponding articles. The analyses were performed on a 500 MHz PC Pentium. Some examples reported during these tests are shown in Figure 4.

Figure 4 shows that the program was capable of elucidating the structures of medium size natural products in just a few seconds. 

## 4. Validation of Performance Using a Large Set of Natural Products 

Testing the system on a large number of problems was deemed to be necessary to obtain statistical data regarding the performance of the application and to accumulate additional experience in the usage of ACD/SE as a CASE tool. This was especially important because the development of the correct method for the application of CASE-based strategies was derived from the application of the software to solve ongoing challenges using real problems. This approach made it possible to detect and overcome multiple hurdles and difficulties which appeared during the development of a practical problem-solving strategy.

In order to obtain sufficient statistical data regarding the results of the system, the structures of 60 natural products reported during a fairly narrow period of time around the period of the study were elucidated [29]. Articles containing relevant experimental data were chosen from issues of the *Journal of Natural Products* mostly published during 2000. As a result, this meant that the structures under study would be absent from the system knowledgebase. During the study molecules containing between 15 and 65 skeletal atoms and with molecular masses ranging from 200 to 900 a.m.u. were studied. Prior to initiating the study, a number of new features, described below, were added to the system to produce a third major iteration of the program, ACD_v3.

*Enhanced predictive capabilities.* ACD/SE_v3 was enhanced to utilize three methods for candidate structure evaluation: (1) a *fast* ^13^C NMR chemical shift prediction based on incremental rules; (2) a more *accurate* ^13^C NMR chemical shift prediction based on the application of HOSE codes [30]; (3) ^1^H NMR chemical shift and, optionally, full spectral prediction including coupling patterns correctly modeling dihedral angle dependencies and second-order effects.

*Smart duplicate removal.* Even while using the constraints imposed by long-range connectivity limits, structure generation can produce identical structures with different carbon atom chemical shift assignments. For this reason, the structural file was checked for the duplication of structures and all but one structure in each family was retained: the one whose ^13^C NMR assignment best matched the calculated ^13^C NMR spectrum and therefore was the “best representative” of the family of duplicates. This process could be performed automatically if the appropriate option was selected. 

*Evaluation of structures*. A two-step structure evaluation procedure was suggested. During the first step *fast* ^13^C NMR spectrum prediction based on the incremental approach [13] was performed for all structures included in the output file. The structures were then rank ordered by increasing *d_F_*, the average deviation between experimental and predicted shifts calculated using the fast prediction approach. The speed of the fast spectral prediction approach would perform calculations at a rate of 1–2 s per structure, even for large molecules, and made it practical to use the approach as an initial screen of all structure candidates, even for lists containing thousands of structures. Many hundreds of studies have shown that the correct structure usually falls very near to the top of the *d_F_*-ranked list.

To better discriminate between the most likely candidates, a more accurate ^13^C NMR spectral calculation (a *fragmental approach*, based on HOSE-code [30]) is performed for the first 10–50 structures of the *d_F_*-ranked list. A second average deviation, the accurate deviation (*d_A_*), was then calculated for each structure. The correct structure is almost always the one possessing the smallest *d_A_* deviation value, which brings the most likely candidate to the top of the list.

To confirm the most probable structure, two additional criteria can be used: (1) For all candidates, ^1^H chemical shifts are predicted, after which the structures are ranked in ascending order according to their average deviations *d_H_*. (2) The ^1^H spectrum can be calculated using the corresponding *J*_HH_ couplings stored in the database. Visual comparison of the predicted spectra with the experimental spectrum can allow the scientist to confirm or reject potential hypothetical structures.

*Utilization of internal and user-created databases.* In some cases, structure generation process can proceed for an extremely long time with no true indication of when it will complete. This can occur when a molecule under investigation is hydrogen deficient, which is quite often observed among compounds related to natural products. In such a situation, the user can initiate a fragment search in the knowledge base containing molecular fragments with assigned chemical shifts from the associated ^13^C NMR spectrum. As a result, the program produces a set of *Found Fragments* (*FF*), which can be integrated into the MCD. This approach allows for significant acceleration of the structure generation process. If a set of assigned structures similar to the molecule under investigation (e.g., derived from the same or similar natural product) is available, ACD/SE provides tools to allow a user to create a *User Fragment Database* (*UFDB*). The latter can be especially useful for a laboratory specializing in a specific area of organic chemistry or chemistry of natural products.

**Figure 5:**
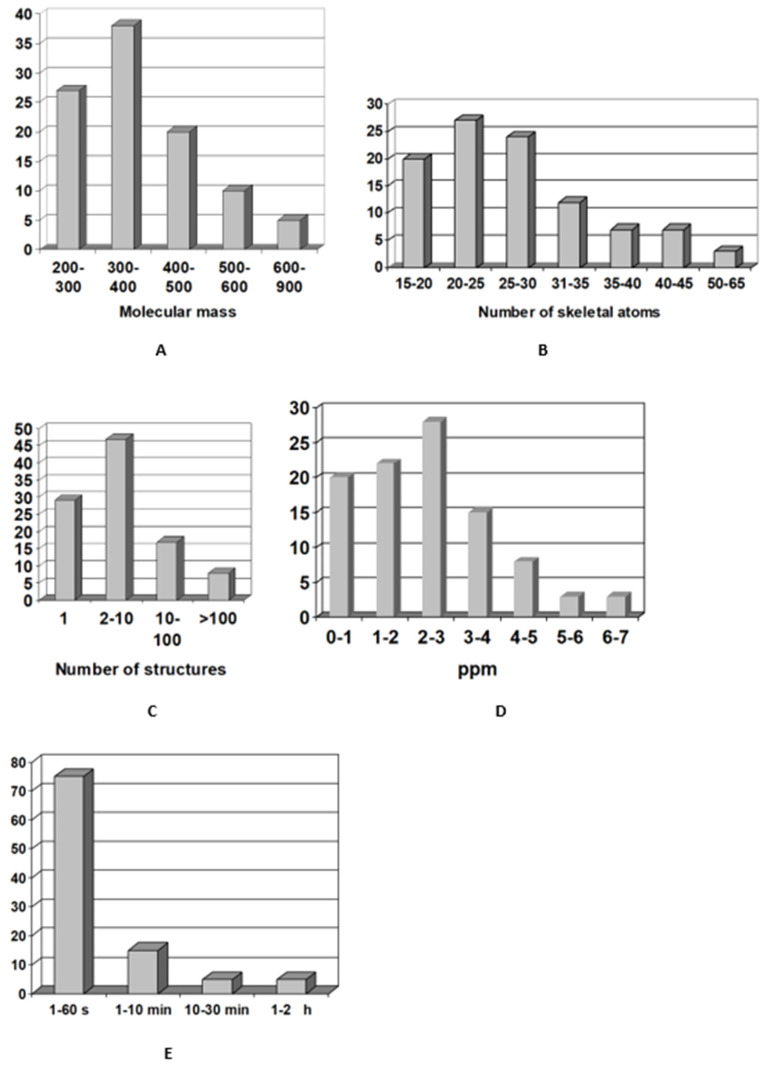
Results of the application of ACD/Structure Elucidator (ACD/SE) to structure elucidation: (**A**) Problem distribution (%) as a function of molecular mass. (**B**) Problem distribution (%) as a function of the number of skeletal atoms. (**C**) Problem distribution (%) with the value of *d_A_* (ppm) calculated for correct structures. (**D**) Problem distribution (%) as a function of the number of structures in the answer file. (**E**) Problem distribution (%) as a function of the time elapsed during structure generation.

Utilization of the user database can also ease structure, elucidation of compounds belonging to the same family. In this study, a first version of an algorithm employing such fragments in ACD/SE were tested. The methodology of applying *FF* for structure elucidation will be considered in detail in Section 7 below. However, the results obtained in the process of elucidating the structures of 60 new natural products were generalized in the histograms shown in Figure 5.

Histogram C (Figure 5) indicates the high selectivity of the system. It shows that the output files contain ten or fewer structures in 75% of the solved problems. Only in 2 of 60 test cases were the correct structures placed in second position by the ranking procedure based on *d_A_* statistics; in all remaining cases it was moved to the top of the list thereby confirming the reliability of the strategy suggested for evaluation of structures delivered by ACD/SE. Moreover, we found that the correct structure occupied the top position in 80% of the solved problems even those cases where preliminary ranking was carried out using *d_F_* values.

It should be noted that high speed and low cost of personal computers makes the issue associated with the time of structure generation inconsequential. Indeed, to extract and purify a new natural product, it can take months and sometimes even years. Therefore, a matter of minutes, hours, or even a few days to elucidate the structure on a PC can hardly be considered unreasonable. Nevertheless, as seen from histogram E (Figure 5), a solution was obtained in one minute for 75% of test cases and the time did not exceed 30 min in 90% of test problems. The research showed that practically all problems requiring more than 10 min were those where only HMBC data were utilized. It is evident that using HMBC and COSY correlations in concert is very desirable and, in some cases, is necessary to provide a solution. 

The results obtained allowed us to conclude that ACD/SE had become a very useful tool for the structure elucidation of complex organic compounds and certainly applicable to solving real world problems that arose in natural product chemistry.

## 5. Identification of Degradants of a Complex Alkaloid 

**Figure 6:**
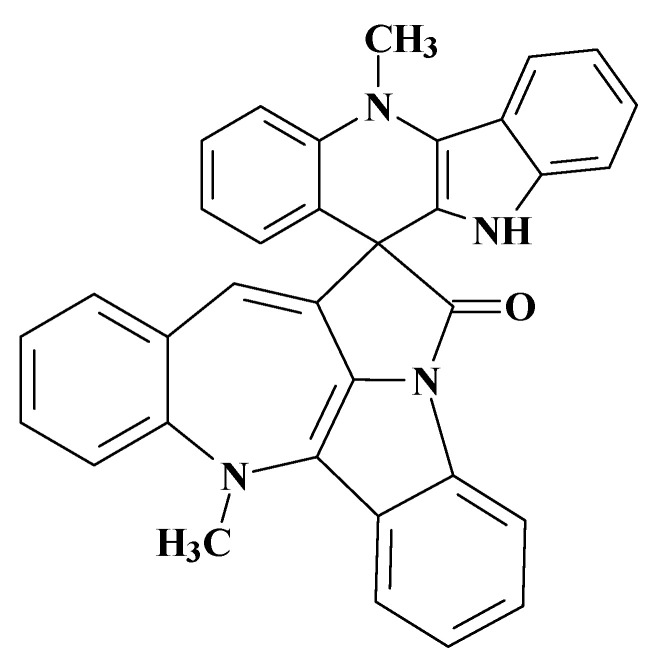
Spiro nonacyclic alkaloid cryptospirolepine **1.**

The first application of ACD/SE to the structure elucidation of a real-world unknown was described in an article resulting from a collaboration between Dr Gary Martin and our group in 2002 [31]. A sample of the complex spiro nonacyclic alkaloid cryptospirolepine (**1**) (Figure 6) that had been stored in a sealed 5 mm NMR tube in d_6_-DMSO for a prolonged period (~10 years) was examined to evaluate the combined utilization of cryogenic NMR probe technology and computer-assisted structure elucidation in the characterization of unknown degradants of this complex molecule. 

The two major degradation products (DP-1 and DP-2) of **1** were isolated by reversed-phase, semi-preparative HPLC. Eluent from the trapping column containing the degradation products was freeze-dried to yield about 1.1 mg of DP-1 at 96% purity by HPLC and about 200 μg of DP-2 at 95% purity by HPLC. NMR samples of about ~0.5 mg and ~200 μg, respectively, were used for the structural characterization effort. Using only GCOSY and GHSQC spectra, the structure of DP-1 was quickly identified as cryptolepinone. This molecule was also found in the ACD/SE knowledgebase, which confirmed the solution.

**Figure 7:**
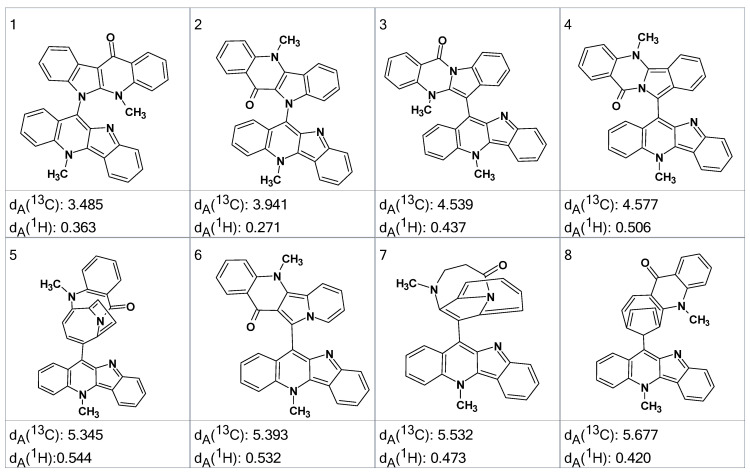
Eight top ranked structures of the output file.

Mass spectrometry on the second most abundant isolate from the degraded sample gave a molecular ion, MH^+^ = 479, which suggested a molecular formula of the DP-2 isolate as C_32_H_22_N_4_O. ^1^H, COSY, HSQC and ^1^H-^13^C HMBC spectra were acquired and input to ACD/SE. Several program runs were carried out to examine the results obtained both with and without user-defined fragments. In the first case, 1268 structures were generated in 10 min and 111 structures were saved after removing duplicates. The eight top ranked structures of the output file are presented in Figure 7.

**Figure 8:**
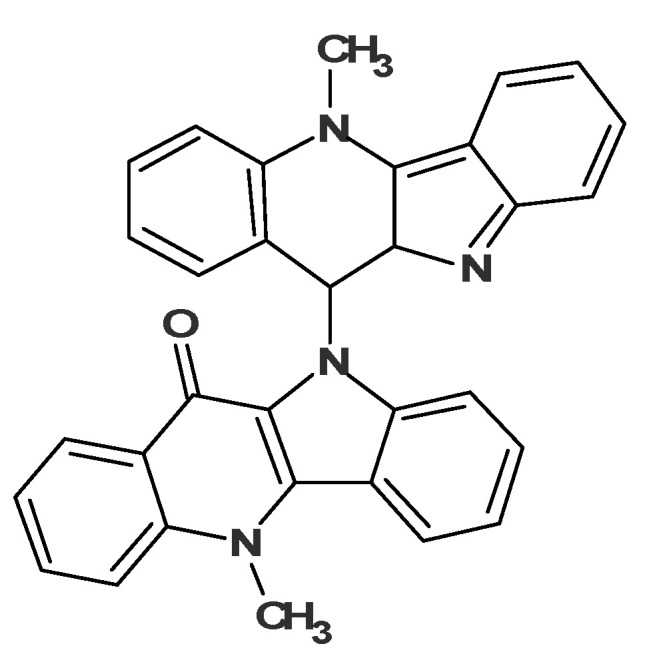
Structure of the degradant **2.**

A ROESY experiment gave four N-methyl to aromatic methine correlations indicating that structure #2 should be selected as a most probable one: all other structures would show either only three correlations of this kind or large *d_A_* and *d_H_* deviations. Therefore, the degradant was identified as compound **2** (Figure 8):

## 6. CASE Outperforming Human Expertise in Structure Elucidation: A First Case

The next ACD/SE application to solving a real-world problem was especially challenging. The problem was posed again by Dr Gary Martin with experimental data supplied by his group. When 2D-NMR data were originally acquired for cryptospirolepine (**1**) in 1991, other data were also accumulated for another alkaloid fraction from *C. sanguinolenta* that was given the notebook designation TC-6. A data set consisting of proton and carbon reference spectra, COSY, ROESY, ^1^H-^13^C HMQC and HMBC spectra in MeOD was acquired. Although it was possible to subgroup the data into four contiguous four-spin systems and to build some slightly larger substructural fragments, a structure consistent with all of the available data could not be assembled in 1991/92 due to a large number of ambiguous connectivities. An attempt was therefore made to elucidate the structure of this alkaloid with the assistance of ACD/SE [32]. The retained reference sample of this alkaloid was found to still be greater than 95% pure by UV (268 nm) peak area with a molecular weight of 448 Daltons. Major fragmentation was simple with the molecule essentially splitting into two “halves” producing fragment ions at 217 and 232 amu. A molecular formula of C_31_H_20_N_4_ was determined from the HRMS.

Despite a relatively congested proton NMR spectrum at 400 MHz, the COSY spectrum still readily allowed the protons of four individual four-spin systems to be identified and ordered. These included ordered sets of resonances as follows:

8.88–8.23–7.76–7.86
8.86–7.59–7.85–7.57
8.68–7.52–7.58–7.11
8.31–7.79–7.53–7.80

**Figure 9:**
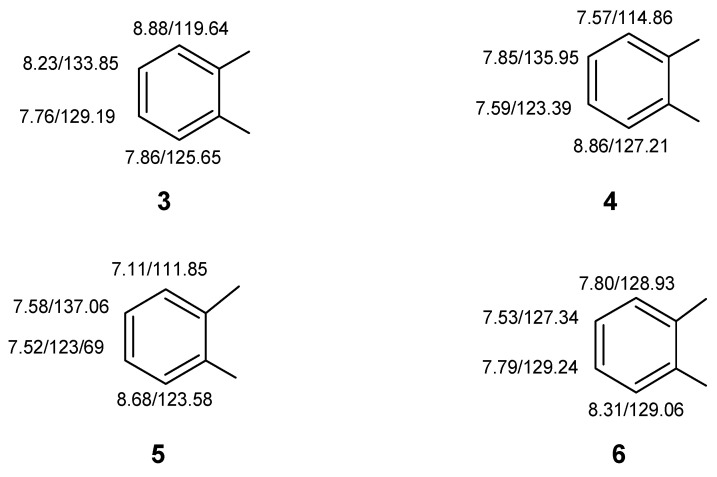
^13^C and ^1^H chemical shift assignments of four-spin systems **3**–**6.**

Using the HMQC correlation data next, the directly bonded carbons were associated with their respective protons giving the substructural fragments illustrated by **3–6** (Figure 9).

**Figure 10:**
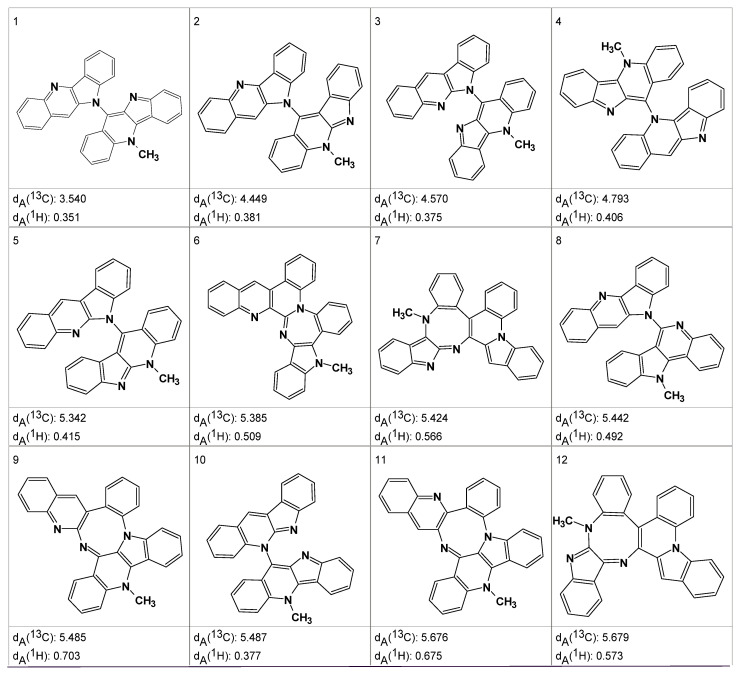
Twelve top ranked structures.

An MCD including these fragments was created. The MCD contained many ambiguous correlations which accounted for the presence of a great number of overlapped peaks in the experimental spectra. Problem solving consisted of the successive removal of ambiguities in the MCD by the user operating in an interactive mode. Approximately 48 h of spectroscopist interaction with the program was required to deliver an MCD in which all ambiguities were resolved. Structure generation from the final MCD was initiated and resulted in the generation of 353 structures, 266 of which were non-isomorphic, after 10 s of computer time. Various spectra were calculated for these structures, including a proton spectrum *d_A_*(^1^H) and both fast [*d_F_*(^13^C)] and accurate [*d_A_*(^13^C)] ^13^C spectra. The structures in the output file were sorted on the basis of *d_A_*(^13^C), and the 12 top ranked structures are shown in Figure 10.

**Figure 11:**
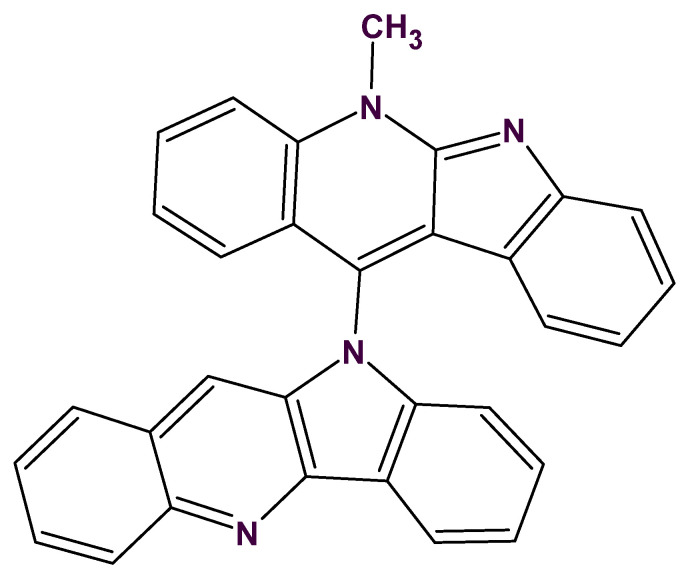
Structure of quindolinocryptotackieine **7.**

Taking into consideration the single observed ROE correlation in the ROESY spectrum from the N-Methyl group as well as the prominent fragment ions at 217 and 232 Da, all structures except #2 may be eliminated. The determined structure **7** (Figure 11) was named quindolinocryptotackieine.

The example described can be considered as a milestone in CASE history as it was the first example of when the program facilitated spectroscopists to elucidate a structure, which had been extremely challenging by manual interrogation of the data.

## 7. Methodology of Applying Found Fragments in ACD/SE

As a result of solving 100 s of problems of increasing complexity, and the resulting benefits of the accumulated experience, we enhanced the algorithms of the ACD/SE application to address those cases where there is a lack of connectivity information characteristic of proton-deficient molecules. In such cases, a fragment library containing about 1,000,000 fragments, as well as user-defined fragments introduced by chemists on the basis of prior knowledge, can be utilized. We provided the ability to embed fragments into the MCDs if they included carbon centers with the appropriate chemical shifts matching the experimental data [33,34,35]. The fragments from the library also include assigned carbon atoms transferred from the molecules used in the construction of the structure library databases. 

Inclusion of fragments into the process of MCD creation is carried out in several stages. First, the program selects a set of *L* Found Fragments as described above (Section 4). The *L* value can practically vary from several hundred to several thousand. At the next stage, an MCD or set of MCDs are created by the program using FFs. In so doing, all FFs, or a limited number of them, can be set by the chemist. The user also set the value of E—the maximum allowed difference between ^13^C chemical shifts assigned to the carbon atoms of fragments and the chemical shifts observed in the experimental spectrum of an analyzed compound. The program compares fragment sub-spectra with experimental chemical shifts, taking into account information regarding multiplicities. Since correlations between experimental ^13^C and ^1^H chemical shifts are observed in 2D NMR spectra, it is necessary to replace those chemical shifts attributed to carbon fragment atoms with suitable experimental chemical shifts to create the MCD. The experimental ^13^C spectrum can often contain several chemical shifts close to the value of a given chemical shift contained within the fragment. The need to exhaustively search all possible options for placing experimental shifts on the atoms of the fragment leads to a complex combinatorial problem, because each arrangement of experimental chemical shifts on the corresponding carbon atoms of a fragment must be verified. If the program establishes that assignments of all carbon atoms are in agreement with the detected 2D NMR correlations, then the corresponding fragment is included in a list of possible fragments.

Obviously, the time associated with structure generation depends on the number of skeletal atoms “absorbed” by the fragments. This time reduces as this number increases. If the program establishes that several fragments correspond with the experimental 2D NMR correlations, then these fragments are “projected” onto the MCDs allowing the user to visually analyze the diagrams and edit them if necessary. Experience shows that the number of MCDs containing found fragments can vary dramatically and, in some cases, can reach several thousand.

**Figure 12:**
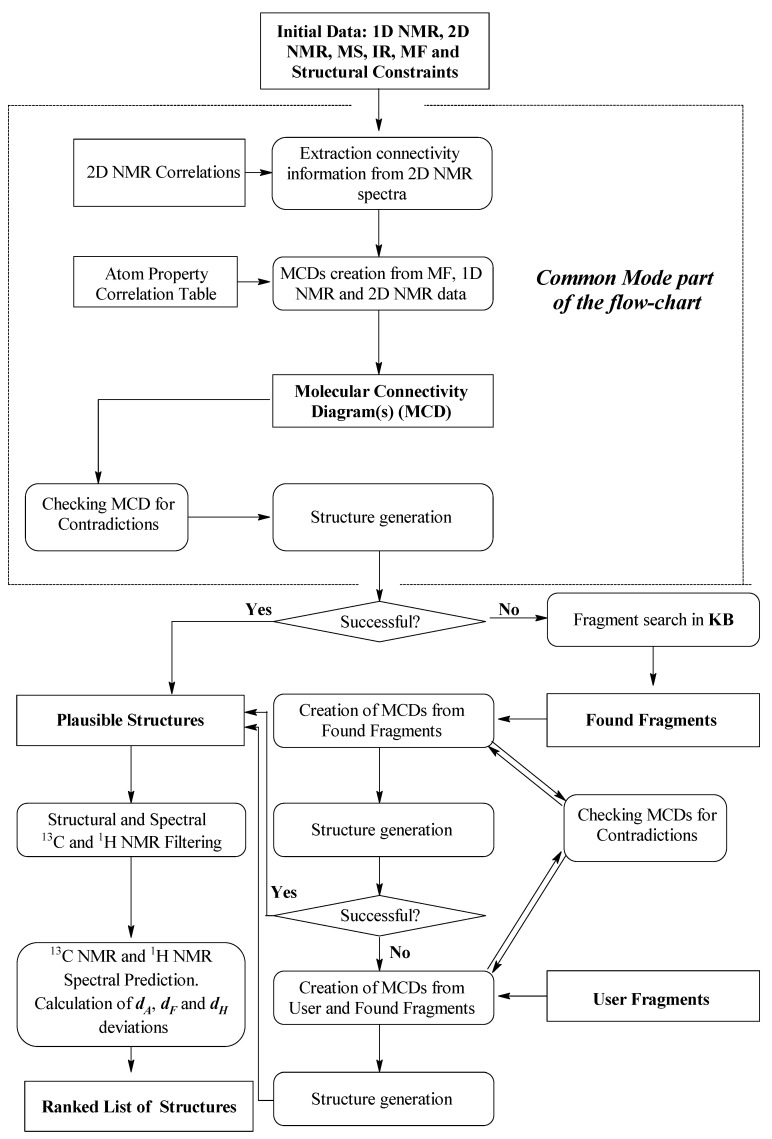
Flow diagram of ACD/SE system: Common and Fragment Modes [33].

A flow diagram of the ACD/SE system where the strategy of structure elucidation uses both the *Common Mode* (without introducing any fragment) and the *Fragment Mode* is presented in Figure 12.

The efficiency of the approach described above was confirmed by testing the application on ca. 150 problems, all based on data related to new natural products and ensuring that their structures were absent from the system knowledgebase. A part of the problem was solved using the Common Mode (without introducing any fragments) and another part of the problem solved using the Fragment Mode. In so doing, a number of especially complicated and challenging problems were identified. Typically, these problems showed that when attempting structure elucidation using the Common Mode, structure generation was so long that it could not be accomplished even after more than 48 h. Attempts to solve these problems using *FF* fragments found in the system knowledgebase also failed. The failure to utilize library fragments was due to the following issues:

- Fragments appropriate for a given problem were missing from the knowledgebase.
- Appropriate fragments are found but the number of possible variants of carbon atom assignments in these fragments is so huge (more than 100 million) that the computer runs out of resources attempting to sort out all possible permutations.
- The number of MCDs built up by the program is so huge that the completion of structure generation is simply too long and human intervention halts the process.

**Figure 13:**
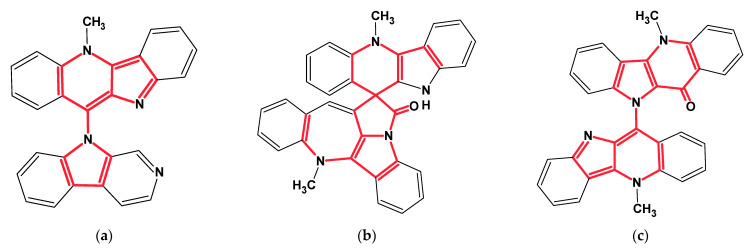
Challenging structures (**a**–**c**) that could not be elucidated using either the Common or Fragment Modes.

Among the structures that we failed to solve in both the Common and Fragment Modes were three alkaloids from the cryptolepine series, presented in Figure 13.

**Figure 14:**
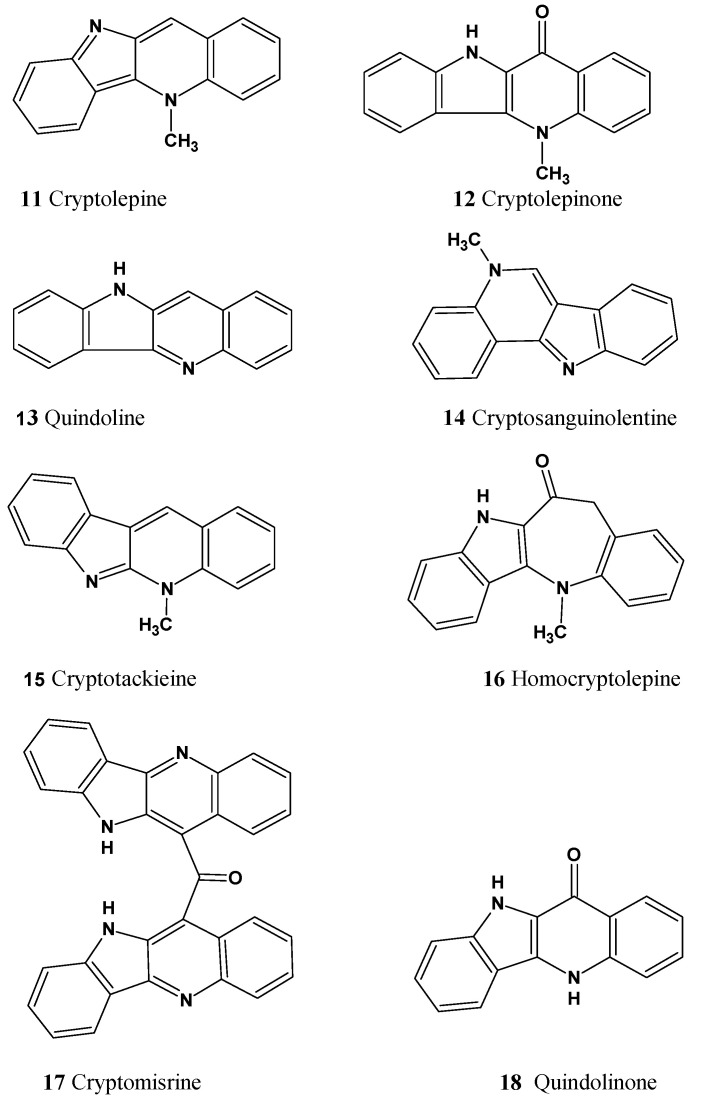
Alkaloids of the cryptolepine family used for creation of the User Fragment Database.

These molecules are relatively large, highly unsaturated, have four condensed benzene rings and double bonds contained in other cycles. All of the molecules have large “silent” fragments (displayed in red) containing no hydrogen atoms. These fragments account for half of the skeleton of the molecule thus limiting the utility of COSY data. For this reason, we considered utilizing a User Database adjusted to analyze the cryptolepine family of structures. Assuming that the unknown compounds were members of the cryptolepine series, we first created a database using earlier published members of the family (see Figure 14).

Structures **11–18,** together with their assigned ^13^C chemical shifts, were used for the creation of a user structure database. Then a User Fragment Database (UFDB) was produced from this database, for which the algorithm common to the ACD/SE system [20] was utilized. Based on specific rules, the program “cut out” a maximum complete set of “chemically reasonable” fragments from each structure. As a result, a UFDB containing 342 fragments from the cryptolepine series was created. 

Utilization of this specialized database allowed us to elucidate structures **8–10**. For instance, 68 fragments were selected as a result of searching the ^13^C NMR spectrum of cryptolepicarboline (**8**) in the UFDB. To minimize the number of free skeletal atoms involved in the structure generation, those options were specified which would provide inclusion of at least three fragments in each MCD. The value of tolerance E was set as 1.5 ppm. The program created 50 MCDs from which 25 survived the test for contradictions. 

Structure generation resulted in: *k* =32→(filtering)→13 (*k* -number of structures generated) and the generation time*, t_g_* is 10 s. NMR spectra prediction positioned the cryptolepicarboline molecule first in the ranked file, where *d_A_*(2) − *d_A_*(1) = 3.3 ppm.

Using the Fragment Mode can dramatically speed up the process of structure generation from 2D NMR data and the use of fragments has frequently transformed very difficult 2D NMR elucidation challenges into easily solvable tasks. This approach can utilize the symbiosis between the spectroscopist and the expert system as the scientist inputs user fragments and participates in the selection of relevant fragments, and this allows problems that seemed to be hopeless at the outset of the structure elucidation process to be solved. Our experiences accumulated as a result of elucidating the structures of 150 natural products were reported in our publications [33,34,35]. 

## 8. Fuzzy Structure Generation 

Since ACD/SE was developed from the very beginning as a commercial system so we tried to supply it with the abilities to solve real world problems which would typically be encountered in academic and industrial laboratories. As a result we placed special attention on overcoming the very serious obstacles associated with the presence of non-standard correlations (NSCs), i.e., HMBC and COSY correlations of *n* > 3 bond lengths, ^n^*J*_CH,HH_.

During the period between 2003 and 2006, we focused on creating a mathematical algorithm to support structure elucidation using 2D NMR data containing an *unknown number of non-standard correlations* and *possessing unknown lengths*. The effectiveness of our work was verified by the elucidation of 50 natural products whose HMBC and COSY spectra contained one or more NSCs [36,37]. *In medias res,* the algorithm was created in the process of solving these problems.

It was demonstrated that ACD/SE could determine the *presence* of NSCs in ca. 90% of all cases using the MCD checking procedure with some NSCs not being detected as the algorithm is based on heuristics. These results were very encouraging since experimental methods guaranteeing the precise determination of COSY and HMBC connectivity lengths are not available. Knowledge of the presence of contradictions in 2D NMR data provides the investigator with valuable information which helps to determine the strategy of structure elucidation with these data. No software message informing the user about the presence of NSCs was provided in these cases. 

For about 50% of cases studied, the program not only identified the contradictions in the data correctly but was able to successfully remove them *automatically* to allow determination of the correct structure. Examples were encountered where the program resolved contradictions caused by the presence of many NSCs (up to a total of 8). In six out of 50 cases, the program was unable to determine the presence of NSCs while in five of those six cases the 2D NMR data contained only one non-standard HMBC connectivity.

The presence of implicit (not detected) NSCs can become apparent as a result of structure generation and filtration: if all of the generated structures obviously contradict the spectral data, the program would most frequently deliver an empty results file. Another indirect evidence of the possibility that contradictions were not detected may be large values, more than 5.5 ppm, of the chemical shift deviation, *d_A_*, calculated for the first ranked structure [29,33]. Investigations have shown that the presence of NSCs was detected by both direct and indirect methods for 95% of the analyzed tasks containing contradictory data.

As was mentioned earlier (Section 3), if NSCs are identified at particular atoms the program automatically lengthens *all* connectivities emanating from such atoms by *one* bond and attempts to generate structures. It is important to emphasize that not all connectivities are lengthened, only the ones identified by the algorithm as potentially being non-standard. However, in those cases when correlations are present in the 2D NMR data with *^n^*J where *n* > 4, this method, unfortunately, does not work. If logical analysis of the MCD failed to detect presence of some implicit NSCs then ACD/SE had no general methodology which would lead to getting solving the problem during the first attempt. To overcome the mentioned drawbacks we developed a more sophisticated algorithm for removing NSCs with the intention of obtaining a valid solution even for these particular situations. In brief, some connectivities are declared by the program as a series to be *suspicious* and are elongated or *eliminated,* and each time structure generation is initiated with a renewed connectivity set. This process we referred to as *Fuzzy* S*tructure Generation* (FSG). 

Let *n* be the total number of connectivities and *m* be the number of connectivities that are suggested to be non-standard. In this case, it is necessary to consider $\left(\begin{array}{c} n\\ m \end{array}\right)=\frac{n!}{m!\left(n-m\right)!}$ different sets from *m* connectivities that will be declared as suspicious. The calculation time increases dramatically as the number of tasks resulting in structure generation sharply increases with the rise of the *m* value.

The FSG algorithm and the strategy for applying it were elaborated in more detail, and tested on a new set of problems [37]. Numerous computational experiments have shown that if the program detects the presence of NSCs but fails to resolve contradictions in the 2D NMR data, then FSG should be used to solve the problem.

Fuzzy structure generation can be easily controlled by parameters that make up a set of options. The two main parameters are: *m*—the number of admitted non-standard connectivities and *a*—the number of bonds by which some connectivity lengths should be augmented. Unfortunately, 2D NMR spectral data cannot deliver definitive information regarding the values of these variables and, as a matter of fact, both of them can be determined only during the process of structure elucidation. We have concluded that in many cases the risk of choosing an erroneous value for *a* can be avoided and the solution of a problem can be considerably simplified if the lengthening of the *m* connectivities is replaced by their *deletion*. When set in the options, the program can ignore them by deleting connectivity responses that have to be augmented (by convention, the parameter *a* is set to a value of 16 in these cases).

Independent of the use of augmentation or removal of connectivities, the crucial point in the application of FSG is the number of connectivity combinations that should be checked during structure generation. For instance, if the total number of connectivities *N* = 60 and *m* = 5 then the number of connectivity combinations, *n_math_* = $C_{N}^{m}$, is equal to ~5.5 million. Any attempt at structure generation has to be performed using each of these combinations. It is necessary to perform generation of structures from each of the $C_{N}^{m}$ data sets and obtain the output file as a unification of all of the intermediate results. Even though the ACD/SE structure generator is fast, the productivity is certainly insufficient in terms of coping with a combinatorial problem such as that outlined here. 

To overcome this difficulty, the program was enhanced with an algorithm capable of *reducing the number of combinations* without the risk of losing the correct solution. This also becomes possible from the results of logical analysis of the initial 2D NMR data. If connectivity sets containing NSCs are identified (see details in [36]), then groups of these connectivities are utilized to produce connectivity combinations. As a consequence, only connectivities that are suspected to be non-standard are included in all resulting combinations and the *initial number of combinations reduces* (as was shown this number could be reduced by several orders of magnitude!). In addition, the algorithm is capable of immediately detecting combinations of connectivities from which structure generation is impossible—a connectivity combination of this kind can still contain at least one NSC. These combinations are skipped during the structure generation process. As a result, FSG can be performed in a reasonable time even in those cases when *n_math_* is very large (~10^10^). It should be noted that given the command “Determine Options Automatically” is activated fuzzy structure generation is carried out in a fully automated mode.

If the MCD checking process fails to detect non-standard correlations in the 2D NMR data (according to our studies the probability of failure is about 10%) the program is forced to try all$\;C_{N}^{m}$ connectivity combinations. This can drastically increase the time to solve the problem and the described approach may be inefficient, but in these cases User Fragments (*UF*) and Found Fragments (*FF*) [33,34] can frequently be helpful. 

To investigate and further optimize the methodology of utilizing FSG, a set of ca. 100 problems were selected [37]. In these problems, either the HMBC or COSY spectra, or both, contained a total of 1 to 18 non-standard connectivities corresponding to a range of coupling constants ^n^*J*_HH_,_CH_ where *n* = 4–6. All structures investigated were natural products and the number of skeletal atoms in the molecules varied between 15 and 75 skeletal atoms. The experimental data were obtained from articles published mainly in the *Journal of Natural Products* or from collaborations with various laboratories. No structure analyzed was present in the knowledgebase of ACD/SE.

Analysis of solutions to the problems showed that all problems could be classified into three sets as follows: 

(1) 53 problems were identified where NSCs were detected, and problems were solved both by updating the initial MCDs and using FSG; 

(2) 34 problems were identified where the program revealed the presence of NSCs but failed to update the MCDs; these problems were solved using FSG;

(3) 13 problems were identified where the program failed to detect NSCs, but they were solved using FSG in the mode when parameter *m* was gradually increased by the user, that is *m* = 1, 2, 3…*m*_0_, where *m*_0_—value at which structure generation is completed with getting a valid solution to the problem.

The results obtained led us to the conclusion that FSG is unique and the most general way of applying CASE to structure elucidation challenges as it is frequently independent of the presence of any number of NSCs with unknown lengths and allows for the identification of the correct structure even in this specific situation. 

### Example

**Figure 15:**
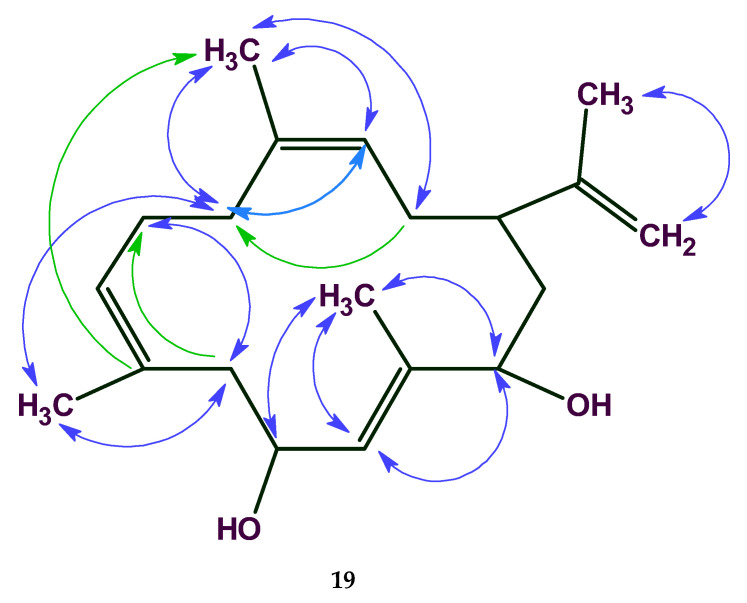
The COSY connectivities are represented on the structure by blue double-headed arrows while the HMBC correlations are defined by green unidirectional arrows from the proton to the carbon to which it is long-range coupled.

In the analysis of cleospinol A [38] with molecular formula C_20_H_32_O_2_ (**19**), the 2D NMR data are comprised of 21 COSY and 55 HMBC correlations. The total number of NSCs presented in COSY and HMBC spectra was equal to 15, while correlation lengths varied from 1 up to 3 (Figure 15). 

The COSY, HMQC, and HMBC spectral data associated with the compound were fed to the program and the MCD was generated. FSG was initiated with the options *m* ≤ 15, *a* = 16.

In this case 18,281,379 connectivity combinations from 40,225,345,056 theoretically possible combinations were created for structure generation. The following result was obtained: *k* = 769→(spectral filtering)→430→(duplicate removal)→245, a generation time of *t_g_* = 29 min 9 s, and the correct structure was ranked first by all methods of spectrum prediction, *r_all_* = 1.

The program therefore identified the correct solution even when 15 non-standard connectivities existed in the 2D NMR data especially in considering that the HMBC and COSY connectivities included both ^6^*J*_CH_ and ^6^*J*_HH_ correlations. Note that only ~10^−4^ of the theoretically possible connectivity combinations were processed. The real number of processed connectivity combinations *n_real_* is more than 18 million. Nevertheless, the high-speed structure generator completed the process in a reasonable time.

## 9. Overcoming the Challenge of Generating Symmetric Molecules

The causes of ACD/SE failing during the structure generation of symmetric molecules from 2D NMR data were carefully investigated and specific peculiarities associated with symmetric structures and their generation were discovered. As a result, an enhanced self-adapting algorithm for structure generation was developed [15]. The algorithm identifies features of molecular symmetry in the 2D NMR data and then adjusts itself to generating symmetric molecules. With this more sophisticated algorithm, the processing time necessary for the generation of symmetric molecules from 2D NMR data is reduced to the order typical for unsymmetrical molecules. Some examples of symmetric molecules elucidated from 2D NMR data are shown in Table 1.

## 10. Determination of Relative Stereochemistry in Small Rigid Molecules

Contemporary structure elucidation commonly includes determination of the relative, and if possible, absolute stereochemistry. For the former, NMR methods are generally well suited, while the latter can require some form of chemical structure modification combined with NMR studies or utilization of X-ray crystallographic methods. Application of NMR data for relative stereochemistry determination is based on the nuclear Overhauser effect (NOE), which is observed from pairs of hydrogen nuclei and is dependent on the distance between them. To provide the data for this analysis in rigid molecules, typically NOESY or ROESY two-dimensional NMR experiments or their selective 1D analogs are used. In order to utilize these data as inputs, ACD/SE was enhanced to allow for the determination of the relative stereochemistry of a small rigid molecule from the constraints deduced from NOESY/ROESY spectra [39]. The results of selective NOE or ROE experiments can also be used as inputs to the program. This procedure can be performed either for the most probable structures inferred by ACD/SE or for a chemical structure proposed by the chemist. In so doing, a direct correlation between the crosspeak volume integration and the internuclear distance is considered. 

Peak intensity in NOE/ROE spectra has an inverse sixth power relationship. The intensities of NOE crosspeaks can be classified depending on the distance *r* between a pair of intervening hydrogens in the following way: r = 1.8–2.5 Å “strong”, r = 2.5–4.0 Å “medium” and r = 2.5–4.0 Å “weak”. If r > 5.0 Å NOE responses are not commonly observed. At the first step, ACD/SE generates all conceivable stereoisomers from a given structure and then selects the most probable one using a minimization algorithm. The latter deals with numerical values extracted from a set of NOEs overlaid on a 3D structure. These values are examined for goodness-of-fit for which a *penalty function* is utilized. The probability of a stereoisomer being a good fit for the data depends on the value of the function: the lower value of the function the better is the solution.

An appropriate penalty function was developed that can be used for verification analysis of *all* stereoisomers, as well as the case when application of a *genetic algorithm* is necessary to limit the number of stereoisomers that need to be investigated. A series of complex structure examples were used to examine the method of relative stereochemistry determination and calculation of 3D models for structures elucidated with the assistance of ACD/SE.

A simpler approach to determine the relative stereochemistry of the molecule [40] was also suggested and examined. The approach is based on ^13^C chemical shift prediction using a fragmental (HOSE code-based) method. We hypothesized that prediction of ^13^C chemical shifts for all stereoisomers generated by the program would allow for preliminary selection of a set of the most probable stereoisomers. The latter could be verified by additional experiments and other computational chemistry methods. This is possible since the algorithm for chemical shift prediction included into ACD/SE is capable of taking into account the presence of stereocenters in the analyzed structure. To verify our hypothesis, we used a series of published examples for novel structures which were deliberately absent from the ACD/CNMR database and whose relative stereochemistry was reliably determined. As a result, it was shown that a limited set of the most probable stereoisomers, which includes the genuine stereoconfiguration, could indeed be selected using the empirical methods of ^13^C chemical shift prediction. Examples are shown in Table 2.

## 11. CASE as an Aid to Avoid Pitfalls during Structure Elucidation

A highly cited review [41] entitled ‘‘Chasing molecules that were never there: misassigned natural products and the role of chemical synthesis in modern structure elucidation” was published by Nicolaou and Snyder in 2005. The authors noted that ca. 1000 articles containing incorrectly determined structures which needed to be revised were published over a period of 15 years (1990–2004).

Figuratively speaking, we can say that 40–45 issues of imaginary “Journal of Erroneous Chemistry” were devoted to publishing only incorrectly determined structures. Consequently, approximately the same number of issues were necessary to describe the revision of these structures. We can expect that the labor costs which are necessary to revise the misassigned structure will be very significant and, generally, can exceed the cost of obtaining the initial structure. As the number of publications in which new natural products are incorrectly assigned is fairly large, the problem of reducing the stream of works containing wrong structures is a valid challenge. 

Nicolaou and Snyder commented that “there is a long way to go before natural product characterization can be considered a process devoid of adventure, discovery, and, yes, even unavoidable pitfalls.”

The Nicolaou and Snyder work [41] prompted us to publish a review [42] where we tried to find answers to the following important questions: (a) Are pitfalls and human errors unavoidable during the process of structure elucidation? and (b) Can modern CASE systems minimize the risk of deducing an erroneous structure from complete and accurately acquired spectroscopic data?

Around 20 examples for which the originally determined structures of novel natural products were revised in later publications were used by us to investigate these questions with the aids of ACD/SE. We tried to determine whether the right structure could be deduced from the initial data including both experimental spectra and assumptions (“axioms”) postulated by the researcher. It turned out that ACD/SE could indeed serve as a tool that is capable of helping the chemist to avoid pitfalls or providing him or her with a cautionary warning. 

The various examples examined in the work led us to conclude that the mistakenly identified chemical structure could be correctly elucidated if 2D NMR data were available and ACD/SE was employed. If even only 1D NMR spectra were measured, then simply the empirical calculation of ^13^C chemical shifts for the hypothetical structures would generally enable a researcher to realize that their structural hypothesis was likely incorrect. We also tried to analyze how erroneous structural suggestions were made by highly qualified and skilled chemists. The investigation of these mistakes is very instructive and has facilitated a deeper understanding of the complicated logical-combinatorial process for deducing chemical structures. This issue was reported on in several publications [43,44,45].

Even though the CASE program is a flexible scientific tool that assists chemists to determine the most likely structure, chemical synthesis clearly still plays an important role in molecular structure elucidation. At the same time, the application of a CASE system would be very helpful even in those cases when chemical synthesis is the crucial evidence to identify the correct structure because a multi-step synthesis requires the confirmation of the intermediate structures at each step, for which spectroscopic methods are commonly used. Experience garnered over years immersed in the field of CASE development led us to the conclusion that utilization of the approach would significantly reduce the number of compounds requiring synthesis to disprove wrong structure and find the correct solution.

## 12. New Fast and Accurate Algorithms for ^13^C and ^1^H Chemical Shift Prediction

Despite the intensive research invested in the field of neural networks (NN) and Partial Least Squares (PLS) regression applications to ^13^C chemical shift prediction a number of issues remained not fully addressed. If they were, this would allow researchers to create faster and more accurate algorithms for NMR prediction and a number of important problems were resolved by our group in 2008–2009. As a result, fast and accurate algorithms of NN and PLS based methods of chemical shift prediction were created [46,47,48]. The algorithms were implemented into ACD/SE, and the performances of both approaches were comprehensively compared. Three methods of NMR shift prediction are now available in ACD/SE: a HOSE code-based method, a significantly improved incremental method and an approach based on artificial neural networks.

The results of chemical shift prediction performed by PLS and NN depend both on the methods of encoding chemical structures into a numerical input and on the associated computational approaches. To provide for more transparent comparison between PLS and NN it is necessary to separate these two parts of the algorithms. These two parts were separately evaluated and compared [46] and a robust and fast PLS algorithm for chemical shift prediction was additionally optimized and examined [47]. We [46] addressed each of the abovementioned issues using ^13^C chemical shifts as the data for analysis. We initially optimized the description scheme used throughout the studies and then the parameters affecting the performance of the neural networks were tuned to achieve optimal performance. Both PLS and neural networks were compared using one of the largest available databases [49] for the purpose of training. The results of the studies were also applied to examine the performance of the ^1^H chemical shift prediction algorithms to demonstrate the general applicability of the developed methods. 

The carbon and proton chemical shift databases used in this work were comprised of approximately 2 million ^13^C chemical shift values. Care was taken to avoid overlap between the datasets used for NN training and algorithm comparison. The *training dataset* was compiled using experimental data published from the early 1990s until 2004. A comparison of the algorithms was performed using a completely independent database compiled from data originally published in 2005 and consisting of approximately 118,000 ^13^C chemical shifts. 

The results of the large scaled computational experiments performed with ACD/CNMR Predictor employing each of the HOSE code-based, NN, and PLS algorithms produced the statistical data necessary for a comparison of performance of all three methods. The corresponding data are collected in Table 3.

The comparison shows that both methods, NN and PLS, can provide results of similar quality after being properly optimized. A neural network, in general, seems to perform better with atoms whose chemical shifts are closer to an average value for the corresponding atomic type. Linear regression can more easily handle exotic fragments since even the most unusual combination of substituents can easily be assigned an appropriate incremental value leading to a more accurate prediction.

ACD/SE is capable of elucidating large molecules containing 100 or more skeletal atoms. Since the initial structural information extracted from 2D NMR data is fuzzy by nature [42] the number of structures that are consistent with the spectral data can usually be rather large (up to tens of thousands [50]). As a result, the selection of the most probable structure from a large output file containing many molecules requires an approach whereby the expert systems can utilize both *accurate* and *fast* approaches for NMR chemical shift prediction. 

The prediction speed was estimated by performing the spectral prediction of thousands of candidate structures generated by the program. It was found that the average speed of the ^13^C chemical shift prediction by PLS is about 10,000 shifts per second on a 2.8 GHz PC computer (about 30,000 on a 3.4 GHz PC), while the neural network-based algorithm was approximately 2.5–3 times slower. The combination of this high speed of prediction with an appropriate accuracy for prediction with an average deviation of 1.71 ppm makes the PLS approach a powerful tool for computer-aided structure elucidation. 

^1^H *prediction*. The experience obtained from the ^13^C-related analysis [46,47,48] was employed to the prediction of ^1^H chemical shifts on the basis of the ACD/Labs NN algorithm. Over 1.2 million ^1^H chemical shifts were used as a training set, and the same test dataset was used as for the ^13^C analysis, a total of ca. 115,000 ^1^H chemical shifts. 

The best results for the ^1^H chemical shifts predictions are presented in Table 4.

Table 4 shows that the neural network and PLS approaches performed in a remarkably similar manner—most of the NN and PLS configurations result in a mean error of approximately 0.2 ppm. In order to reduce the error to that experienced in experimental determinations further optimization such as the detailed description of the 3D geometry might be necessary.

After updating and improving the aids of NMR chemical shift prediction, the following standard procedure of the most probable structure selection was established.

*First step*. ^13^C NMR spectra are predicted for all generated structures using the fastest incremental method, and *d_I_* values are calculated. 

*Second step*. Duplicate structures in the output file are deleted. In so doing *d_I_* values of each duplicate family are considered and, as described above, only the structure that has the minimum *d_I_* value is retained in the file as the “best representative” of the family.

*Third step*. ^13^C chemical shift prediction is carried out using a NN algorithm and the structural file is ranked again with *d_N_* deviations. If the resulting file is extremely large the calculations can be applied only to the first several thousand structures. As a result of this step the preferable structure is selected with greater reliability.

*Fourth step*. During the fourth stage, ^13^C NMR spectra are usually calculated for the first 10 to 50 structures of the ranked file using fragmental method based on the HOSE code approach. The average deviation values between the experimental and calculated values (*d_A_*) are found and the structures are again rank-ordered. Subsequent ranking increases the probability of moving the correct structure to the first position in the list. For additional control over the correct choice of the output structure, the HOSE code-based proton chemical shifts can be predicted and displayed together with the corresponding deviation value *d_H_*. As a rule, the final structural ranking is carried out according to increasing *d_A_* and *d_N_* values, while magnitudes of the *d_I_* and *d_H_* parameters serve as secondary aids for estimating reliability of the correct structure selection.

## 13. Elucidation of “Undecipherable” Structures

As discussed earlier, at the beginning of the 2000s we used ACD/SE to elucidate the structure of a complex alkaloid whose identification turned out to be a particular challenge even for experienced spectroscopists. Over a decade later, in the early 2010s, we analyzed data associated with several small and, at first glance, simple molecules that puzzled the experts. However, ACD/SE was able to resolve these puzzles [51,52].

**Figure 16:**
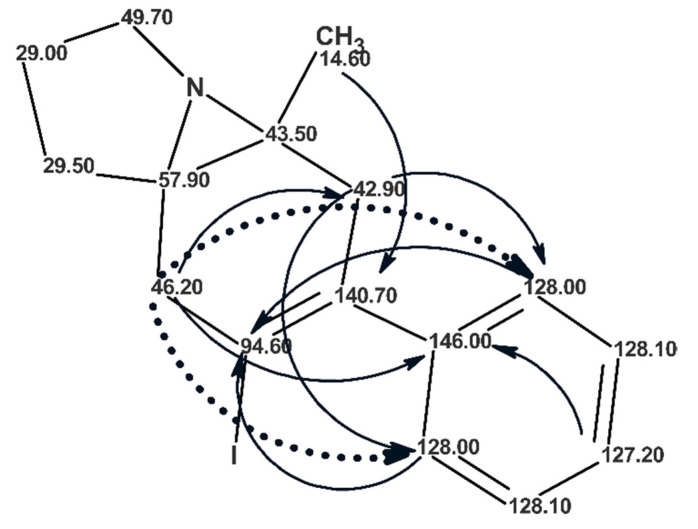
Suggested structure of a reaction product. The arrows show the nine ^1^H-^13^C HMBC NSCs. The connectivities from 46.2 to 128.0 marked by dotted arrows correspond to ^5^J_CH_.

Kummerlöwe and co-workers [53] investigated a product with a molecular formula of C_16_H_18_NI that was obtained by reacting an azide-containing 1,5-enyne in the presence of electrophilic iodine sources. Combined use of IR, MS, 1D ^1^H and ^13^C, COSY, HSQC, ^1^H-^13^C HMBC, ^1^H-^15^N HMBC and 1,1-ADEQUATE experiments did not allow the authors [53] to elucidate the structure. The problem was solved by using residual dipolar couplings (RDC’s) and it was one of the first examples of applying RDC to elucidate a small molecule structure. The authors [53] suggested a set of candidate structures for the unknown and tested them using experimental RDCs to identify the correct structure. The structure elucidated, **20**, is shown in Figure 16.

Figure 16 shows that the ^1^H-^13^C HMBC spectrum of **20** contains nine NSCs (including two *intense* ^5^*J*_CH_ cross peaks marked by a dotted line), which was the main cause precluding structure elucidation using a traditional approach: the initial set of “axioms” was extremely contradictory. The molecular formula, 1D-^13^C, HSQC, ^1^H-^13^C HMBC and ^1^H-^15^N HMBC data [53] were entered into ACD/SE [51]. No assumptions or user interventions were used. As a result of Fuzzy Structure Generation, three structures were generated in 13 min and the correct structure was placed in first position by the ranking procedure. When 1,1-ADEQUATE correlations were also added to the 2D NMR data only one correct structure was generated in 0.7 s.

**Figure 17:**
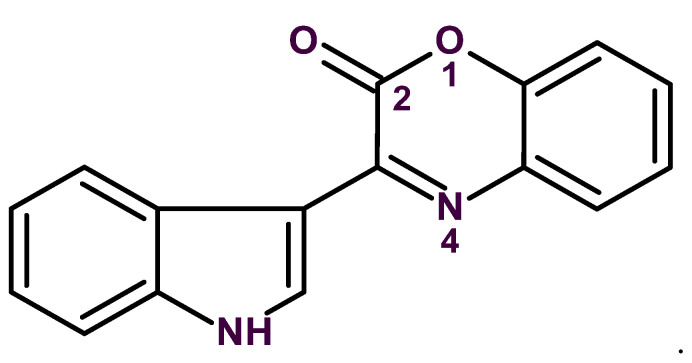
Structure of the natural product cephalandole A (**21**).

Gross and coworkers [54] were the first who suggested to employ Atomic Force Microscopy (AFM) [55] for determining a challenging structure of a small planar molecule by making the image of its skeleton visible. In order to validate the method’s utility, they studied the known natural product cephalandole A, (**21**, Figure 17), with the molecular formula C_16_H_10_N_2_O_2_.

The molecule meets criteria that render structure analysis especially challenging: the ratio of skeletal atoms to hydrogen atoms is equal to 2:1, and the O and N atoms at positions 1 and 4, respectively, interrupt the carbon skeleton completely, separating the two parts of the molecule. 

**Figure 18:**
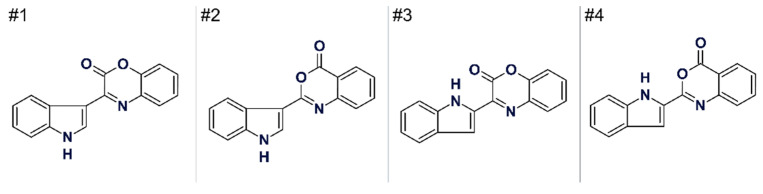
Possible structures of cephalandole A proposed by Gross and co-workers [55].

On the basis of 1D and 2D NMR data analysis the authors [55] suggested four conceivable structures (see Figure 18). The authors concluded that the available NMR data did not allow the distinction between a 2- or 3- substituted indole substructure, and therefore all four structures could be considered plausible. The first structure is the accepted structure of cephalandole A, and the second one is the previously misassigned structure of this compound. 

Gross and co-workers [55] have demonstrated that the AFM approach, combined with quantum-chemical computations helps to identify the first structure as the most probable one by comparing AFM molecular images with structures presented in Figure 13. The suggested method gives researchers a new independent tool to distinguish between planar molecules that have similar structures. 

The 1D and 2D NMR (HSQC, COSY and HMBC) spectra acquired by the authors [55] to analyze this problem were entered into ACD/SE. As contradictions were detected in the spectroscopic data, FSG was run and the program produced an output file of 11 structures in 1 m 50 s. The first structure in Figure 13 was selected by the ranking procedure as the most probable candidate, while the second structure was ranked 9th (i.e., rejected by the program). It is noteworthy that the other two proposed structures in Figure 13 were not generated at all. This work showed that AFM requires a set of conceivable structures on which molecular images would be imposed in order to identify the final candidate. 

In a follow-up study [52] a combination of AFM with CASE, 1D and 2D NMR spectra and DFT calculations was used for the first time to determine the structure of breitfussin A, a natural product isolated from the Arctic hydrozoan *Thuiaria breitfussi* (Family Sertulariidae). The molecular formula of the unknown was found to be C_16_H_11_BrN_3_O_2_. With a Double Bond Equivalence of 12, and a ratio of skeletal atoms to hydrogen atoms of 2, the structure of breitfussin A is highly proton deficient. A small amount of sample meant that X-ray diffraction analysis was not possible.

**Figure 19:**
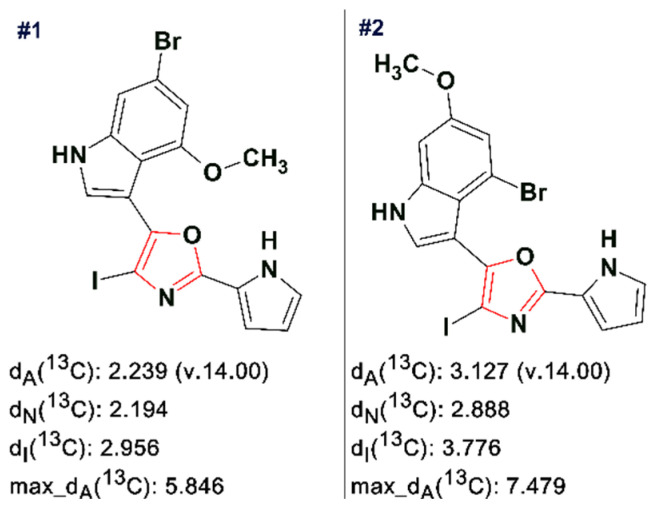
The two best structures of the ranked output file. The part of the structure highlighted in red is deprived of hydrogens.

The HMBC and COSY spectra of breitfussin A were entered into ACD/SE. To minimize the potential risk that the correct structure would not be elucidated, generation was run with very few constraints, producing 4,545,009 structures, 505 of which were saved after filtering, for a total of 48 h 37 m computing time. The ranking procedure selected the two best structures presented in Figure 19.

**Figure 20:**
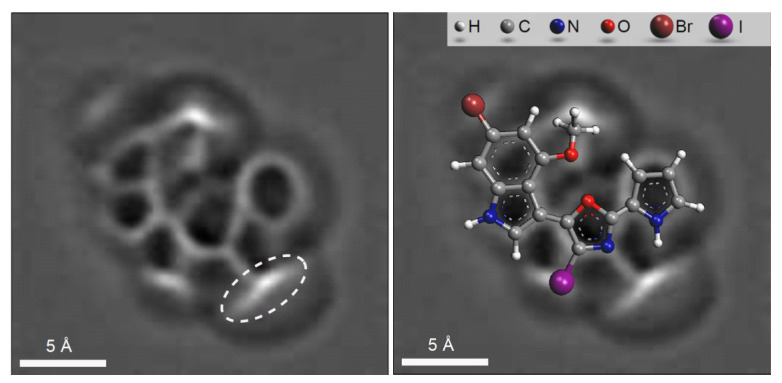
Molecule image (**left**) and superposition of the structure suggested by CASE on the image (**right**). The white encircled region marks an image artifact. This allowed the reliable assigning of the correct structure of breitfussin A. A posterior analysis showed that the ^1^H-^13^C HMBC spectrum contained 11 NSCs (ten of them 4-bonds in length and one 5-bonds in length), which would have rendered the problem insolvable manually if only NMR data are used.

An AFM image of a single molecule of the unknown was superimposed on both the selected structures (see Figure 20), and it turned out that the image coincided with the structure #1 in Figure 19. 

## 14. DFT-Enhanced Method for the Best Structure Selection

**Figure 21:**
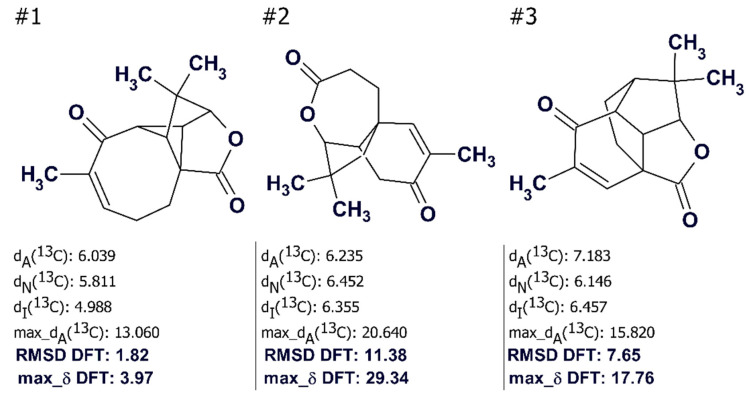
Ranked output file for aquatolide produced by ACD/SE. RMSD DFT is based on the difference between the experimental and DFT calculated ^13^C chemical shifts, and the max_δ DFT is the maximum deviation the between experimental and DFT calculated ^13^C chemical shifts.

Experience indicates [14] that if the ACD/SE output file contains even ca. 100,000 structures, the utilization of empirical methods of chemical shift prediction [49] and structure ranking elevates the most likely structures to the top of the file. If several top ranked structures are characterized by close deviations, then the solution to the problem will be ambiguous. A method to resolve the ambiguity was suggested by Buevich and Elyashberg [56,57,58]. It has been reported [56,57] that the uncertainty in distinguishing the correct structure can be overcome by a DFT calculation of chemical shifts (and coupling constants if necessary) for the 4–6 top-ranked structures of the output file. For example, during the structure elucidation of aquatolide, ACD/SE produced three structures (Figure 21) and the first ranked structure #1 coincided with that of aquatolide that had been suggested earlier [59]. However, Figure 21 shows that the average deviation values *d_A_, d_N_,* and *d_I_* are significantly higher than those typically observed in similar analyses (*d_n_* < 3 ppm, *n* = A, N, I) and therefore selection of the best structure was deemed to be suspicious. 

When the ^13^C chemical shifts of the three structures were calculated using the DFT approach the priority of the structure #1 was reliably confirmed by the values of RMSD and the max_δ calculated by the QM method (see Figure 21). 

**Figure 22:**
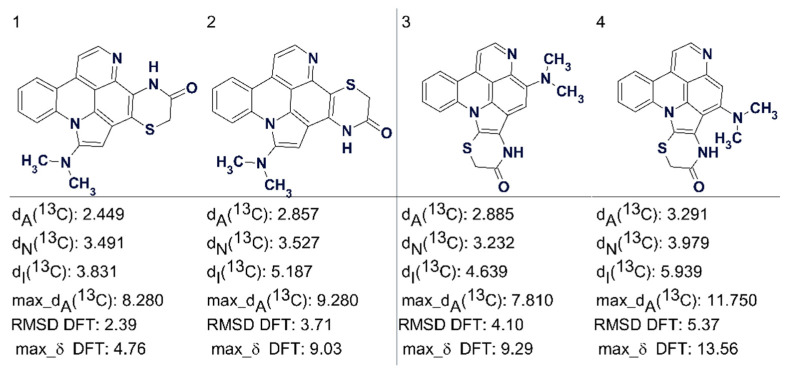
Top ranked plausible structures suggested by the ACD/SE program for cycloshermilamine D. Notations are similar to those listed in Figure 21.

Another example is that of cycloshermilamine D, where the highest ranking structures suggested by CASE are shown in Figure 22 [57]. Here all the deviations are of acceptable but very similar values. Figure 22 shows that DFT calculations confirmed the validity of the first structure.

Atom coordinates and chemical shifts calculated by DFT methods can be imported into ACD/SE and therefore available to complement the empirical methods. 

In a follow-up work [58] the application of DFT methods in CASE methodology was further expanded. The authors explored the possibility of applying DFT predictions of ^n^*J*_CH_ and ^n^*J*_HH_ couplings to confirm the correct structure selected by the ACD/SE program that utilizes the Fuzzy Structure Generation algorithm. It has been shown that DFT-predicted *J*-couplings associated with non-standard correlations can serve as an orthogonal parameter to the chemical shifts in the verification of the correct structure. The attained results also fully justified the validity of non-standard correlations that were utilized in the FSG protocol in an ad hoc manner. 

The author’s conclusions were confirmed by the analysis of NSCs in eleven real-world natural products elucidated by ACD/SE. Additionally, utilizing the example of the CASE study of cleospinol A (Figure 15), it was shown that the DFT computed *J*-couplings of NSCs can distinctively differentiate the correct structure among six proposed isomers. The proposed approach of NSC verification should further improve the robustness of CASE analysis and can help reveal potential problems with reported experimental data. We believe that the addition of DFT calculations of *J*-couplings to the CASE expert systems should further improve the robustness of the CASE algorithm and thus help to expend its application to ever more complex molecular structures. 

## 15. New Enhancements Implemented into ACD/SE

### 15.1. Application of ChemSpider and PubChem Databases for Dereplication

Dereplication is the process of testing samples that are active in a screening process, with the intention of recognizing and eliminating from consideration those substances that have already been characterized [60]. The process is directed by a minimal set of analytical data inputs used to search across a database of known materials. Such inputs generally include molecular formula (generally obtained by accurate mass measurement), λ_max_ from UV-Vis spectroscopy, chemical shifts from (generally 1D-) NMR, and molecular fragments evident from NMR spectroscopy and mass spectrometry. Developing methods of natural products dereplication using large databases which contain millions of diverse chemical compounds has great potential. 

Such methods are becoming increasingly important because as the number of reported natural products increases, it is vital to have an efficient method for directing discovery efforts. This is especially true in drug discovery efforts where the expense of *de novo* isolation and structure elucidation of known compounds is prohibitive. The most common method of dereplication is the use of mass spectrometry, MS. However, MS-based methods can lead to the misidentification of compounds due to differences in ionization and multiple molecular entities having the same molecular formula. Therefore, elaboration of an approach allowing to carry out dereplication using aids of ACD/SE looked rather attractive. 

A strategy for the dereplication of a complete or a partial structure using ^1^H NMR, 1H-^13^C HSQC and ^1^H-^1^H COSY spectral data, a molecular formula composition range and structural fragments against a ChemSpider database snapshot of about 22 million compounds (at the time of the study) was implemented into ACD/SE. The effectiveness of the approach was confirmed using a series of examples. Furthermore, the methodology developed was successfully applied to natural product dereplication using the PubChem database which contained 110 million compounds at the time of study.

### 15.2. Speeding up Structure Generation

Despite recent advancements expert systems are still susceptible to a series of limitations which also impede structure elucidation by a human expert. These limitations are mainly associated with the ambiguity of the experimental data. Ambiguity can also be due to the hybridization state of carbon nuclei that appear in the same regions of NMR spectra. For example, a ^13^C NMR signal observed at 90 ppm can belong either to a *sp*^2^ or *sp*^3^-hybridized carbon connected to one or two oxygen atoms. If a molecule contains atoms that can have variable valences (e.g., N and/or P), then all of their possible valences should be explored during structure generation.

In such cases, the only remaining solution is the exhaustive investigation of all alternatives resulting from the presence of any ambiguity. For example, if there are five carbon nuclei with signals in the range of 70–120 ppm, then 32 combinations of hybridization (*sp*^3^ or *sp*^2^) have to be generated and checked. This could significantly increase the structure generation time. To speedup structure generation in the presence of ambiguities of the various types mentioned, the structure generation algorithm was reworked so that multi-threading calculations could be used [61]. Depending on the constraints displayed in the MCD generation time could be decreased by the factor of 2–10 as a result of this optimization.

### 15.3. Determination of Relative Stereochemistry and 3D Models

The method of relative stereochemistry determination discussed earlier, based on the utilization of a genetic algorithm, did not find wide application as it required that the user be capable of setting up the appropriate options for the genetic algorithm. An alternative method which does not require any user special mathematical knowledge was developed and utilizes NOESY/ROESY spectra in combination with the RDKit [62] program to perform conformational analysis.

As explained earlier NOESY or ROESY spectra usually produces correlation peaks for ^1^H atoms within a 5 Å distance. This criterion is normally sufficient to define the stereochemistry of either a small rigid molecule or one with no more than two or three stereocenters. If the structure is flexible, or has more stereocenters, it is necessary to carefully evaluate the intensity (via the integrals) of the NOE or ROE crosspeaks in order to get a more quantitative idea about the actual distances between the involved nuclei. While the theoretical intensity of a NOESY/ROESY crosspeak depends on the inverse 6th power of the distance between the two nuclei, the actual measured intensity depends on the experimental parameters used (mixing time) as well as relaxation and molecular motion.

The suggested approach for the determination of the best stereoisomer includes the following steps: (a) the generation of all stereoisomer structures corresponding to a given structural formula of a molecule, (b) conformational analysis of all stereoisomers with distance constraints extracted from the NOESY/ROESY spectra, (c) selection of the best stereoisomer based on the minimal distance RMSD and minimum energy. The details of the approach are described in [63].

## 16. Conclusions

ACD/Structure Elucidator has been developed over two decades to produce a high performance computer-assisted structure elucidation (CASE) software application that has elucidated thousands of chemical structures. The application has been improved incrementally over the years and now utilizes the most complete suite of capabilities delivered in any CASE system to solve chemical structures exemplified by complex natural products with many tens of skeletal atoms. The application uses algorithms for the integrated handling of data from 1D NMR spectra for multiple nuclei and a suite of multiple forms of 2D NMR spectra. Data associated with hundreds of thousands of chemical structures have been used to train multiple types of NMR predictors spanning from fast to accurate prediction. Similar large libraries of chemical structures have been used to produce a fragment library containing over a million fragments that are used during the elucidation process. These data continue to expand with time as new complex structures are added into the libraries. ACD/SE is presently installed in many academic and industrial laboratories across the world and provides support to scientists on a daily basis who are challenged with elucidating complex structures. While development continues unabated the foundation developed over the past twenty years, and represented in this article, represents the dedicated efforts of an enormous team of scientists, mathematicians, NMR spectroscopists and software developers who have produced this application. We, as authors, are proud to represent the work of the team that developed Structure Elucidator during this period.

## Figures and Tables

**Table 1:** Examples of symmetric molecules elucidated by ACD/SE. Here *n* is the total number of skeletal atoms and m is the number of NSCs.

#	Structure	Protocol	*t_g_*
1	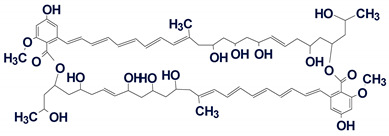 C_68_H_92_O_18_, *n* = 86	HMBC COSY FSG at *m* = 2 Result: *k* = 96→6	3 m
2	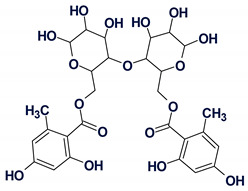 C_28_H_34_O_17_, *n* = 45	HMBC COSY FSG at m = 4. Result: *k* = 28,180→36	4 m
3	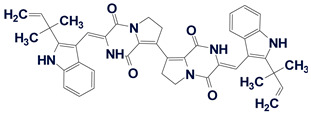 C_42_H_40_N_6_O_4_, *n* = 52,	HMBC COSY Common Mode Result: *k* = 720→21	35 m
4	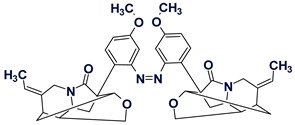 C_40_H_44_N_4_O_6_, *n* = 50	HMBC COSY Common Mode Result: *k* = 384→93	47 s

**Table 2:** Examples of structures for which sets of preferable stereoisomers were selected using empirical methods of ^13^C NMR chemical shift prediction.

Example No.	Structure	Number of Stereo Isomers	Position of Correct Stereoisomer
1	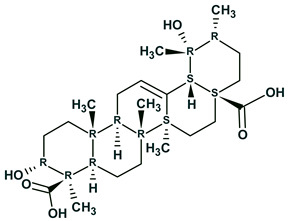	1024	1
2	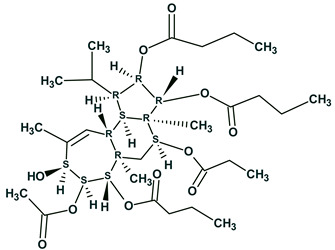	1024	3
3	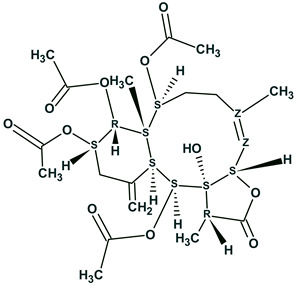	256	8

**Table 3:** The results for the prediction of both ^13^C chemical shifts by HOSE codes, PLS and NN algorithms. Outliers are defined as those shifts predicted with an error of more than 10 ppm.

Prediction Method	Mean Error, ppm	Standard Deviation, ppm	Maximum Error, ppm	% of Outliers
HOSE	1.8	3.05	58.0	2.8
PLS	1.7	2.61	51.6	0.7
NN	1.6	2.45	85.8	0.6

**Table 4:** The results for the prediction of ^1^H chemical shifts by HOSE codes, PLS and NN algorithms. Outliers are defined as those shifts predicted with an error of more than 1.0 ppm.

Prediction Method	Mean Error, ppm	Standard Deviation, ppm	Maximum Error, ppm	% of Outliers
HOSE	0.19	0.30	3.94	1.3%
PLS	0.18	0.26	2.72	0.7%
NN	0.18	0.26	3.71	0.8%

## Data Availability

Not applicable.
